# The gene for a lectin-like protein is transcriptionally activated during sexual development, but is not essential for fruiting body formation in the filamentous fungus *Sordaria macrospora*

**DOI:** 10.1186/1471-2180-5-64

**Published:** 2005-11-03

**Authors:** Minou Nowrousian, Patricia Cebula

**Affiliations:** 1 Lehrstuhl für Allgemeine und Molekulare Botanik, Ruhr-Universität Bochum, 44801 Bochum, Germany

## Abstract

**Background:**

The filamentous fungus *Sordaria macrospora *forms complex three-dimensional fruiting bodies called perithecia that protect the developing ascospores and ensure their proper discharge. In previous microarray analyses, several genes have been identified that are downregulated in sterile mutants compared to the wild type. Among these genes was *tap1 *(*t*ranscript *a*ssociated with *p*erithecial development), a gene encoding a putative lectin homolog.

**Results:**

Analysis of *tap1 *transcript levels in the wild type under conditions allowing only vegetative growth compared to conditions that lead to fruiting body development showed that *tap1 *is not only downregulated in developmental mutants but is also upregulated in the wild type during fruiting body development. We have cloned and sequenced a 3.2 kb fragment of genomic DNA containing the *tap1 *open reading frame and adjoining sequences. The genomic region comprising *tap1 *is syntenic to its homologous region in the closely related filamentous fungus *Neurospora crassa*. To determine whether *tap1 *is involved in fruiting body development in *S. macrospora*, a knockout construct was generated in which the *tap1 *open reading frame was replaced by the hygromycin B resistance gene *hph *under the control of fungal regulatory regions. Transformation of the *S. macrospora *wild type with this construct resulted in a *tap1 *deletion strain where *tap1 *had been replaced by the *hph *cassette. The knockout strain displayed no phenotypic differences under conditions of vegetative growth and sexual development when compared to the wild type. Double mutants carrying the Δ*tap1 *allele in several developmental mutant backgrounds were phenotypically similar to the corresponding developmental mutant strains.

**Conclusion:**

The *tap1 *transcript is strongly upregulated during sexual development in *S. macrospora*; however, analysis of a *tap1 *knockout strain shows that *tap1 *is not essential for fruiting body formation in *S. macrospora*.

## Background

Fruiting body formation in ascomycetes is a complex process leading to the formation of a number of specialized cell types from a comparatively undifferentiated vegetative mycelium [1]. Recently, the molecular basis of this process has been investigated by forward and reverse genetics approaches, and a number of genes that are essential for fruiting body development have been identified. However, a coherent picture of fungal multicellular development has yet to emerge [2].

One avenue towards a deeper understanding of developmental processes is by functional genomics analyses, e.g. microarray studies. Such approaches can help to identify genes that are regulated differentially during fruiting body development and are therefore candidates for further functional analysis. In a previous study, we have performed microarray analyses of fruiting body development in *S. macrospora *[3]. This ascomycete is homothallic and produces fruiting bodies called perithecia within seven days under laboratory conditions. *S. macrospora *is a close relative of *N. crassa*, but in contrast to *N. crassa*, it does not produce any asexual spores. Therefore, changes of gene expression patterns during sexual development are not superimposed by changes related to asexual sporulation. We have previously analyzed gene expression in three developmental mutants of *S. macrospora*, and have identified a number of genes that are downregulated in the sterile mutants when compared with the wild type [3]. One of these genes is *tap1 *(*t*ranscript *a*ssociated with *p*erithecial development, formerly known as *SMU5651 *[3]). *tap1 *transcript levels are downregulated in the three developmental mutants pro1, pro11 and pro22 as well as in all three double mutants which led us to speculate that the gene might be involved in sexual development in *S. macrospora*. In addition to this intriguing expression pattern, the derived TAP1 amino acid sequence shows homology to lectins from other filamentous fungi with the highest similarity to lectins isolated from fruiting bodies of several basidiomycetes [3]. It has long been speculated that lectins play a role in fungal development; however, as no mutants in lectin-encoding genes from fruiting body-producing fungi have been analyzed to date, definite proof for this hypothesis is lacking [4,5]. The only known fungal lectin mutant is a strain of *Arthrobotrys oligospora *in which the lectin gene *aol *was deleted [6]. This mutant does not exhibit any phenotypical differences from the wild type under all conditions investigated, but as no sexual cycle is known for *A. oligospora*, the question whether *aol *might be involved in fruiting body development could not be addressed. Here, we present data on the expression of the *S. macrospora tap1 *gene as well as the characterization of a *tap1 *knockout strain to address the role of a putative lectin in fungal sexual development.

## Results

### Expression of *tap1 *during sexual development in *S. macrospora*

**Figure 1:**
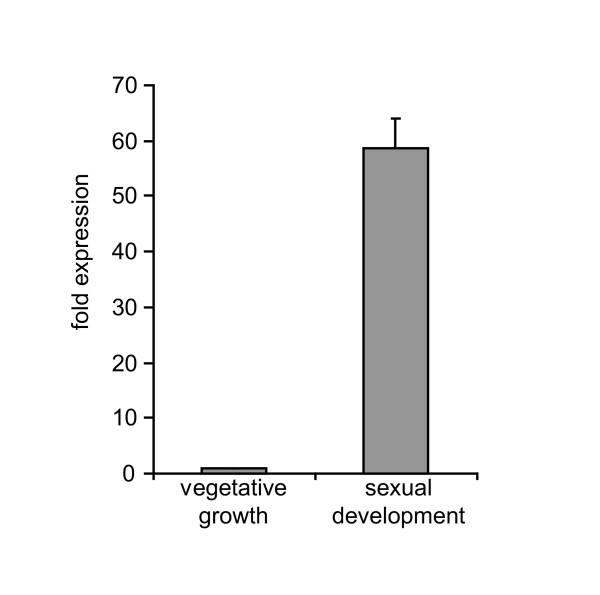
**Transcript levels of *tap1 *are upregulated during sexual development. ***tap1 *transcript levels in the wild type under conditions of vegetative and sexual development were analyzed by real time PCR. For normalization, transcript levels of the SSUrRNAs were used as described previously [3]. Expression is given as fold induction (mean of two independent experiments, error bars indicate standard deviation) with transcript levels during vegetative growth set to 1. Real time PCR results were tested for significance of differential expression at a level of p = 0.001 using REST [24].

Previously, we reported that *tap1 *is downregulated in several developmental mutants when compared with the wild type [3]. In that analysis, all strains were grown under conditions that allowed sexual development, i.e. in floating culture. To investigate whether *tap1 *expression in the wild type is associated with sexual development, we compared transcript levels of *tap1 *in sexually developing mycelia with vegetative mycelia. For this purpose, the wild type was grown in floating or in submerged culture where it develops either fruiting bodies or vegetative mycelium, respectively. Analysis of *tap1 *expression by quantitative real time PCR revealed that *tap1 *transcript levels are upregulated nearly 60-fold in mycelia undergoing sexual development (Figure 1). These data further confirm that *tap1 *expression is linked with fruiting body development in *S. macrospora*.

### Sequence analysis of the *S. macrospora tap1 *gene

**Figure 2:**
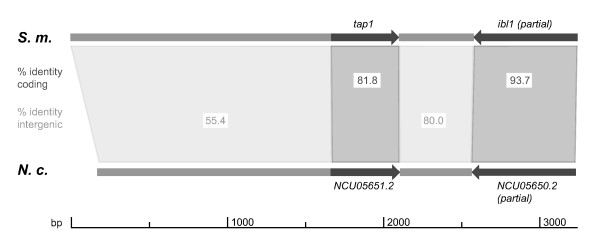
**Synteny between *S. macrospora *(*S.m.*) and *N. crassa *(*N.c.*) in the genomic region containing *tap1*. **Coding regions are given as dark gray and intergenic regions as light gray boxes. Nucleic acid identities between the *S. macrospora *open reading frames and their *N. crassa *orthologues as well as between the intergenic sequences are indicated.

For previous expression analyses, part of the *tap1 *open reading frame was amplified from *S. macrospora *genomic DNA by PCR [3]. For further characterization of *tap1*, we have isolated a *tap1*-containing cosmid clone from an indexed cosmid library of *S. macrospora *[7]. A 3.2 kb restriction fragment carrying the *tap1 *open reading frame and adjacent regions was subcloned and sequenced. The fragment carries the uninterrupted *tap1 *open reading frame of 429 bp encoding a predicted polypeptide of 143 amino acids. In addition, the fragment contains part of another open reading frame downstream of *tap1 *that was named *ibl1 *(*i*mportin *b*eta *l*ike 1) due to its similarity to an importin beta subunit. The sequenced region is syntenic to a region within the *N. crassa *genome that contains the open reading frames *NCU05651.2 *and *NCU05650.2 *that are orthologs of *tap1 *and *ibl1 *respectively (Figure 2). In a previous comparison of 85 protein-coding genes of *S. macrospora *and *N. crassa*, the average nucleic acid identity within coding regions was found to be close to 90 %, and even non-coding regions of these two closely related Pyrenomycetes can be readily aligned [8]. These findings are further supported by our analysis of the genomic region containing *tap1 *(Figure 2).

**Figure 3:**
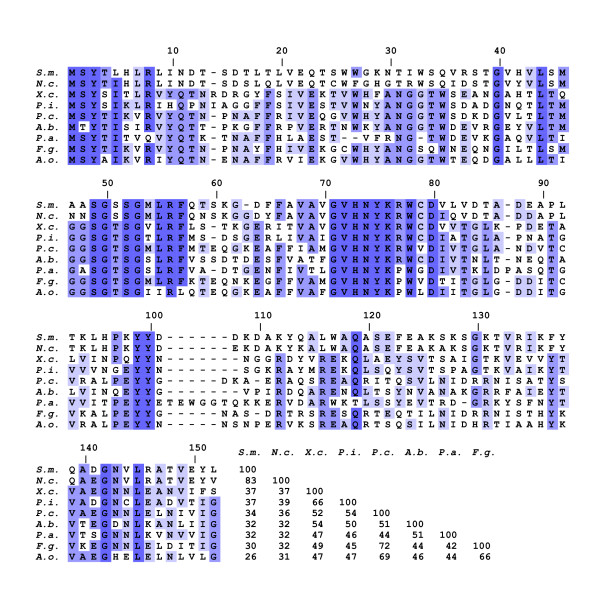
**Multiple alignment of TAP1 and lectins from filamentous fungi. **The multiple alignment was created using CLUSTALX [25] with the following sequences: *S.m.*, *Sordaria. macrospora *TAP1 [this work, emb:CAH03681.2]; *N.c.*, *Neurospora crassa *NCU05651.2 [ref:XP_325506.1]; *X.c.*, *Xerocomus chrysenteron *lectin [gb:AAL73236.1]; *P.i.*, *Paxillus involutus *LECA [gb:AAT91249.1]; *P.c.*, *Pleurotus cornucopiae *PCL-F2 [dbj:BAB63923.1]; *A.b.*, *Agaricus bisporus *ABL [sp:Q00022]; *P.a.*, *Podospora anserina *Pa5D0092 [emb:CAD60779.1]; *F.g.*, *Fusarium graminearum *FG07558.1 [gb:EAA76455.1]; *A.o.*, *Arthrobotrys oligospora *lectin [emb:CAA65781.1]. Jalview was used to visualize the alignment [26]. Amino acid residues conserved in at least eight of the nine sequences are given in dark blue, residues conserved in at least six sequences in medium blue, and residues present in at least four sequences in light blue. At the end of the alignment, amino acid identity in % is given for all sequences in pair-wise comparisons.

The predicted TAP1 polypeptide was used for BLASTP [9] searches of the public databases, and a multiple alignment was constructed of TAP1 and several of its closest homologs from other fungi (Figure 3). The closest homolog is the predicted protein NCU05651.2 from *N. crassa*; however, interestingly, the second-best sequence identity is found in two lectins from the basidiomycetes *Xerocomus chrysenteron *and *Paxillus involutus*. Surprisingly, lectins and predicted lectin-like proteins from the ascomycetes *Podospora anserina*, *Fusarium graminearum*, and *A. oligospora *are less similar to the *S. macrospora *TAP1 even though these fungi are much more closely related to *S. macrospora *than the basidiomycetes (Figure 3). However, the (predicted) lectins from *P. anserina*, *F. graminearum*, and *A. oligospora *have a higher similarity compared to the basidiomycete lectins than TAP1 does (Figure 3). These findings might indicate that TAP1 and its corresponding *N. crassa *ortholog, even though being clearly members of this fungal lectin family, have evolved rather fast compared to other ascomycete homologs.

### Construction of a Δtap1 strain

**Figure 4:**
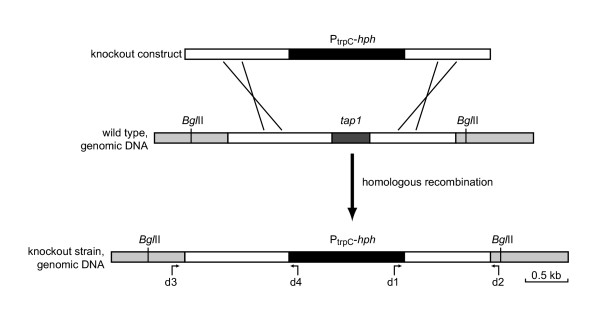
**Strategy for the generation of a *tap1 *knockout strain. **The *tap1 *open reading frame is shown in dark gray, flanking non-coding regions that are present in the knockout construct are given as white boxes, and adjoining regions not present in the knockout construct are shown in light gray. The Hygromycin B resistance cassette comprising the *hph *gene and the *trpC *promoter and 5' UTR is given in black. Positions of oligonucleotide primers d1 to d4 are indicated. For further information see text.

**Figure 5:**
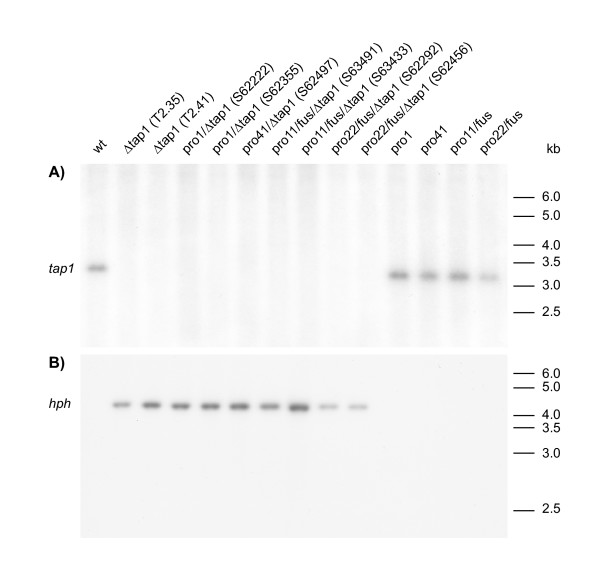
**Southern blot analysis of Δtap1 strains. **Genomic DNA from the wild type, from two different Δtap1 single spore isolates from the original knockout strain, and from double mutants of Δtap1 with pro1, pro41, pro11, and pro11 was hydrolyzed with *Bgl*II, and after gel electrophoresis, the Southern blot was probed with radio-labeled DNA fragments containing the open reading frames of *tap1 *(A) and *hph *(B), respectively. Marker sizes in kb are provided on the right. Numbers in brackets are strain numbers for single spore isolates.

A construct for generating a *tap1 *deletion strain was generated. It consists of a hygromycin resistance cassette flanked by about 1 kb of sequences upstream and downstream of the *tap1 *open reading frame (Figure 4). The construct was used to transform the *S. macrospora *wild type, and transformants were screened for homologous recombination by PCR with oligonucleotide pairs d1 and d2 as well as d3 and d4 (Figure 4). These primer combinations yield amplicons only in the case of homologous recombination. Among 50 primary transformants, one transformant was found that displayed the expected PCR fragments (data not shown). Ascospores from this transformant were isolated to purify the putative knockout strain, and Southern blot analysis of nine ascospore isolates was performed with probes for *tap1 *and the Hygromycin resistance gene *hph*. For two of the ascospore isolates, T2.35 and T2.41, results are shown in Figure 5. As expected, the *tap1 *probe hybridized to a 3.2 kb *Bgl*II fragment in genomic DNA of the wild type, whereas no hybridization signal was obtained with genomic DNA from the knockout strains T2.35 and T2.41. Conversely, the *hph *probe marked a 4.2 kb *Bgl*II fragment in the knockout strains and produced no signal with wild type DNA. The 4.2 kb fragment is of the size expected in a strain where homologous recombination has taken place (Figure 4). The Δtap1 strains T2.35 and T2.41 were used for further analysis.

### Phenotypic characterization of the Δtap1 strain in different genetic backgrounds

**Figure 6:**
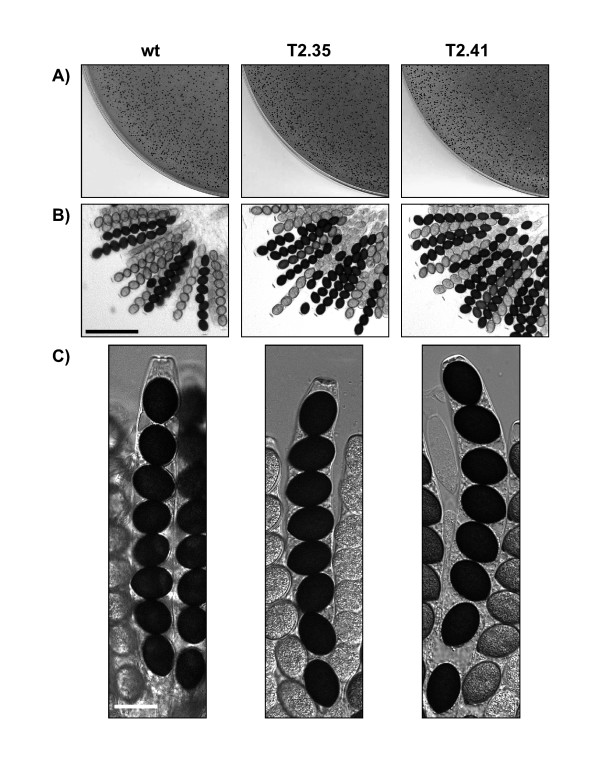
**Δtap1 strains have a wild type-like phenotype. **The wild type and Δtap1 single spore isolates T2.35 and T2.41 (as indicated above each column) were grown in Petri dishes on BMM solid medium [10, 21] for 6d (A) or 7d (B, C) at 25°C in constant light. A) Segments of petri dishes, black dots are individual fruiting bodies. B) Segments of ascus rosettes with mature (black-spored) and immature asci. Scale bar 100 μm. C) Mature asci. Scale bar 20 μm.

The *tap1 *deletion strains T2.35 and T2.41 were analyzed with respect to their phenotype during the sexual cycle. Surprisingly, both were completely wild type-like in all aspects of fruiting body development, i.e. both *tap1 *deletion strains produced mature perithecia in the same numbers and in the same amount of time as the wild type. Moreover, neither perithecial nor spore morphology were altered in any recognizable way (Figure 6). Fruiting body formation took place both in constant light as well as in constant darkness both on complete as well as minimal media, similar to that of the wild type. Ascospores from the knockout strains germinated readily and were black like wild type spores (Figure 6).

We then investigated whether there were any phenotypes unrelated to sexual development present in the mutant strains. Mycelial growth rates were determined as 24.7 (± 1.7) and 24.8 (± 1.4) mm per day for Δtap1 and wild type respectively; thus, indicating that mycelial growth is not altered in the knockout strains. Also, renewal of growth and fruiting body development after incubation at 4°C or 37°C, conditions that prevent growth and fruiting body formation, respectively, was not different in the mutants when compared to the wild type (data not shown). Also, wettability of mycelium and fruiting bodies was similar in wild type and mutants indicating that the hydrophobic coating of the mycelium was not altered in the knockout strains. As the lack of *tap1 *did not cause any discernable phenotype, we wondered whether *tap1 *might have a redundant function or whether its absence might be masked by the presence of other genes. We therefore crossed the Δ*tap1 *allele into strains bearing mutations in other developmental genes, namely *pro1*, *pro11*, *pro22*, and *pro41*. The pro1 mutant is defective in a gene encoding a transcription factor [10], the pro11 mutant lacks a functional WD40 repeat protein [11], and pro22 and pro41 are mutants non-allelic to *tap1 *or the other *pro *genes (Kück et al., unpublished data). *tap1 *transcript levels are downregulated in mutants pro1, pro11, pro22 [3], and pro41 (Nowrousian, unpublished data). Thus, we speculated whether a complete lack of *tap1 *would lead to a more pronounced developmental phenotype, especially as several other developmental genes are also downregulated in the pro mutants [3]. We obtained double mutants with strains pro1, pro11, pro22, and pro41 and verified the presence of the Δ*tap1 *allele in the double mutants by Southern blot analysis (Figure 5). All double mutant strains were phenotypically similar to the respective single pro mutants in that they produced only protoperithecia and were therefore sterile. There were no differences in the number of protoperithecia produced by the double mutants when compared with the single mutants. Also, growth rates of the vegetative mycelium as well as overall morphological appearance were the same (data not shown). For the crosses of the tap1 knockout with the pro mutant strains and subsequent back-crosses, we analyzed a total of 17 full tetrads and 37 partial tetrads from crosses with different mutant strains and in all cases found the expected 1:1 segregation pattern for each of the single markers (hygromycin resistance and pro mutant phenotype, respectively). This is a further indication that deletion of *tap1 *does not interfere with sexual development, and also shows that the process of generating the Δ*tap1 *allele did not introduce further mutations into the strains that would cause any different segregation patterns. Overall, no phenotype for Δ*tap1 *was found in any of the genetic backgrounds investigated.

## Discussion

The *tap1*-encoded polypeptide from *S. macrospora *has significant homology to lectins and lectin-like protein from other fungi (Figure 3). The highest degree of amino acid identity is found in comparison with lectins that were isolated from fruiting bodies of several basidiomycetes [12-14]. Lectins are carbohydrate-binding proteins that are found in a variety of organisms [4,5]. On the basis of sequence homology, the *S. macrospora *TAP1 polypeptide can be included into a class of fungal lectins; however, whether it has lectin activity, i.e. whether it specifically binds carbohydrates, remains to be elucidated. Interestingly, TAP1 displays a greater sequence identity towards basidiomycete lectins than to lectins or putative lectins from ascomycetes with the (notable) exception of its closest homolog, the *N. crassa *protein NCU05651.2 (Figure 3). However, BLAST searches in the *N. crassa *genome [15] with the sequences of TAP1 as well as the lectin sequences from the other fungi used in our sequence comparisons yielded only NCU05651.2 as a significant result (data not shown). This finding indicates that the gene is present as a single copy in *N. crassa*, and that there is no other member of this lectin gene family present in the *N. crassa *genome. As *N. crassa *and *S. macrospora *are close relatives with highly syntenic genomes [8], it is likely that this is the case in *S. macrospora *as well. This observation is supported by the fact that only a single band in *S. macrospora *genomic DNA hybridizes with a *tap1 *probe (Figure 5). Thus, it seems that this particular class of fungal lectins has evolved faster in *S. macrospora *and *N. crassa *compared to other ascomycetes, for which it still retains more similarity with its basidiomycete relatives (Figure 3). Another class of fungal lectins has been found in the basidiomycete *Coprinus cinereus*. These lectins bind galactose and are therefore called galectins, and two galectins from *C. cinereus *are specifically expressed during different stages of fruiting body formation [16,17]. However, BLAST searches for galectin homologs in the *N. crassa *genome yielded no significant results (data not shown) indicating that no galectin-like proteins exist in *N. crassa *or that their sequences are too dissimilar to the *C. cinereus *sequences to be detected by sequence comparisons alone. Thus, the *N. crassa *NCU05651.2 gene and its *S. macrospora *ortholog *tap1 *are so far the only genes encoding putative lectins that have been identified by sequence analysis in these two ascomycetes.

In fungi, most lectins have been isolated from basidiomycetes, especially from mushroom fruiting bodies, and it has been speculated that they play a role in fruiting body development [5,18,19]. However, as no lectin mutant in a fruiting body-producing fungus has been characterized to date, this hypothesis has not been verified experimentally. Previous investigations and the results presented here show that *tap1 *transcript levels are closely correlated with fruiting body development in *S. macrospora*; therefore, we decided to construct a *tap1 *knockout strain to analyze whether this putative lectin plays a role in fruiting body formation. A Δtap1 strain was generated by gene replacement, but the knockout strain has no discernable phenotype under all conditions investigated. This might indicate that *tap1 *has indeed no function in vegetative growth or sexual development of *S. macrospora*; however, the possibility that *tap1 *is needed under environmental conditions not tested in our experiments or that its function is redundant or that in the absence of *tap1*, another gene product can take its place, cannot be excluded. The latter effect is well known in other organisms, and it was, for example, tested in a large-scale analysis of yeast synthetic lethal interactions where it was found that genes involved in similar biological processes, but not necessarily in the same regulatory pathway, can buffer one another in single mutant backgrounds but show a phenotype in the double mutant strain [20]. To test whether any of the known developmental genes of *S. macrospora *show this kind of genetic interaction with *tap1*, we obtained double mutants of Δtap1 with strains bearing mutations in the developmental genes *pro1*, *pro11*, *pro22*, or *pro41*. However, all double mutant strains were phenotypically similar to the respective pro mutant strains. *tap1 *transcript levels are downregulated in the pro mutants, and our analysis demonstrates that even the complete loss of *tap1 *does not worsen the condition of the mutants. This leaves open the possibility that *tap1 *is necessary in a different genetic background or under different environmental conditions.

With respect to fruiting body formation, our results show that the putative lectin-encoding gene *tap1 *is not an essential gene for this developmental process. As mentioned previously, the only other known fungal lectin mutant is the aol mutant of the nematode-trapping fungus *A. oligospora *[6]. Similar to our findings, the aol mutant also has no phenotype under all conditions investigated. Since no sexual stages from *A. oligospora *are known, these observations do not include fruiting body formation; however, vegetative growth, conidiation, and nematode-trapping were unchanged in the aol mutant strain [6]. Thus, possible functions of this class of lectins and lectin-like proteins in filamentous fungi remain enigmatic.

## Conclusion

*tap1 *expression is strongly associated with sexual development in *S. macrospora*. An analysis of the *tap1 *gene and its surrounding genomic region revealed a high degree of sequence identity and overall synteny with the corresponding region in the genome of *N. crassa*. Sequence comparisons of TAP1 with lectins and lectin-like proteins from other fungi indicate that it is most closely related to lectins isolated from basidiomycete fruiting bodies. However, analysis of a *tap1 *knockout strain shows that *tap1 *is not essential for fruiting body formation nor vegetative growth in *S. macrospora*. This is the case for a *Δtap1 *allele in an otherwise wild type genetic background as well as in combination with mutations in several developmental genes. Whether *tap1 *has any function under growth conditions not investigated here, e.g. in a more natural setting, remains to be elucidated.

## Methods

### Strains and growth conditions

Details of the *S. macrospora *strains used in this study are provided in Table 1. Double mutants were obtained from crosses of single mutant strains. Double mutant genotypes were verified by crosses against single mutants. Unless stated otherwise, standard growth conditions and DNA-mediated transformation were as previously described [10,21]. For analysis of growth velocity, 30 cm long race tubes were filled with 15 ml of medium, inoculated at one end and the growth front was marked every 24 h for 7 consecutive days. For RNA extraction from cultures developing fruiting bodies, *S. macrospora *was grown at 25°C in constant light in floating culture as described [3]. For RNA extraction from vegetative mycelium, a mycelial plug of 0.7 cm in diameter from a Petri dish with liquid medium [3] was inoculated into an Erlenmeyer flask with 100 ml of liquid medium and shaken at 130 rpm.

### RNA extraction and quantitative real time PCR

Extraction of total RNA and quantitative real time PCR were performed as described previously [3] with the following modifications: reverse transcription was performed with 400 U Superscript II (Invitrogen) and 0.33 mM dNTPs, and real time PCR was carried out in a DNA Engine Opticon 2 (MJ Research).

### Identification of a cosmid clone carrying *tap1 *and analysis of the *tap1 *gene

An indexed *S. macrospora *cosmid library [7] was screened for *tap1 *by PCR with oligonucleotides SMU5651-1 (5' CATCAACGACACCTCCGACACCC) and SMU5651-2 (5' CATCGGCCTGATAGAACTTGATCC). For a first round of screening, pooled DNA from 48 cosmid clones was used as a template, DNA from clones from positive pools was then subpooled and used for the next round of screening. This led to the isolation of cosmid D3 from pool VI518-614 that contains the *tap1 *gene. A 3.2 kb *Bgl*II restriction fragment carrying *tap1 *was subcloned from cosmid D3 into pBluescript II/KS+ (Stratagene). The insert of the resulting vector pPC24 was sequenced at MWG Biotech or GATC Biotech AG [emb:AJ781427.2].

### Construction of a Δtap1 strain

To create a *tap1 *knockout construct for homologous recombination in *S. macrospora*, flanking regions upstream and downstream of the *tap1 *open reading frame were amplified by PCR from *S. macrospora *genomic DNA using oligonucleotides SMU5651-BamHI (5' AGGATCCGTGATTCTCATGCTGTGGAAGGAAGC) and SMU5651-NheI (5' AGCTAGCTTTGGCGGTTTGGTTGGGGGGTTGGT) for the upstream region and oligonucleotides SMU5651-ApaI (5' AGGGCCCGTACTCGTCAGTGGGAAAGTGGGTGG) and SMU5651-SacI (5' AGAGCTCTATGCACTTGCTCCTCAAGCGTCTC) for the downstream region, introducing restriction sites as indicated in the oligonucleotide names. Additionally, the hygromycin-resistance cassette consisting of the *hph *gene from *Escherichia coli *and the *trpC *promoter from *Aspergillus nidulans *was amplified from plasmid pCB1004 [22] using oligonucleotides Hph-ApaI (5' AGGGCCCTCAACGGAACCCTATTCCTTTGCCC) and Hph-NheI (5' AGCTAGCAACTGATATTGAAGGAGCATTTTTGG). All PCR fragments were subcloned in pDrive (Qiagen) resulting in pDriveA (upstream region), pDriveB (downstream region) and pDrivehph (hygromycin-resistance cassette). Sequences of inserts and orientation within the vector was verified by restriction analysis and sequencing (MWG Biotech AG). The PCR fragment containing the downstream region was obtained by *Apa*I/*Sac*I digestion of pDriveB and cloned into *Apa*I/*Sac*I digested vector pDriveA resulting in plasmid pDriveAB. The hygromycin-resistance cassette was obtained by digesting plasmid pDrivehph with *Nhe*I and *Apa*I and was cloned into plasmid pDriveAB hydrolyzed with *Nhe*I and *Apa*I resulting in the knockout plasmid pABXY. For transformation of *S. macrospora*, plasmid pABXY was digested with *Bam*HI and *Sac*I and the knockout cassette was obtained by gel elution. The knockout cassette was transformed into the *S. macrospora *wild type and primary transformants were screened for homologous integration by PCR. For this purpose, total DNA was prepared from the transformants according to [23], and PCR was performed with oligonucleotides d1 (5' CGATGGCTGTGTAGAAGTACTCGC) and d2 (5' TGCCTCCTCCGAGGCTGATAACCT) for the downstream region or d3 (5' CGGTGGGTAAGGTATCTCTGATG) and d4 (5' CACCGCCTGGACGACTAAACCAA) for the upstream region (Figure 4).

## Authors' contributions

MN designed the study, performed expression analyses and isolation of the cosmid clone as well as part of the characterization of the knockout strain and wrote the manuscript. PC cloned and sequenced the *tap1 *gene, made the knockout construct and the *tap1 *knockout strain and carried out part of the characterization of the knockout strain.

## Figures and Tables

**Table 1:** *S. macrospora *strains used in this study. All strains are single spore isolates and are kept in our laboratory collection. The *fus *allele that is present in some of the strains is a spore color marker (brown instead of black spores) but has no influence on growth or fertility.

strain number	genotype	description
S48977	wild type	wild type
T2.35	Δtap1	tap1 deletion strain
T2.41	Δtap1	tap1 deletion strain
M8871	pro1	sterile mutant [10]
S24117	pro11	sterile mutant [11]
S22528	pro22	sterile mutant
S46357	pro41	sterile mutant
S62222	Δtap1, pro1	sterile mutant
S62355	Δtap1, pro1	sterile mutant
S62497	Δtap1, pro41	sterile mutant
S63491	Δtap1, pro11, fus	sterile mutant
S63433	Δtap1, pro11, fus	sterile mutant
S62292	Δtap1, pro22, fus	sterile mutant
S62456	Δtap1, pro22, fus	sterile mutant
